# Contamination Pattern and Risk Assessment of Polar Compounds in Snow Melt: An Integrative Proxy of Road Runoffs

**DOI:** 10.1021/acs.est.2c05784

**Published:** 2023-03-02

**Authors:** Loïc Maurer, Eric Carmona, Oliver Machate, Tobias Schulze, Martin Krauss, Werner Brack

**Affiliations:** † Department of Effect-Directed Analysis, UFZ—Helmholtz Centre for Environmental Research, Permoserstr. 15, 04318 Leipzig, Germany; ‡ Institute of Ecology, Evolution and Diversity, Goethe University, Max-von-Laue-Str. 13, 60438 Frankfurt am Main, Germany

**Keywords:** chemicals of emerging concern (CECs), liquid chromatography high resolution mass spectrometry (LC-HRMS), snow melt, urban road runoffs, risk assessment, tire wear compounds, pesticides, pattern analysis

## Abstract

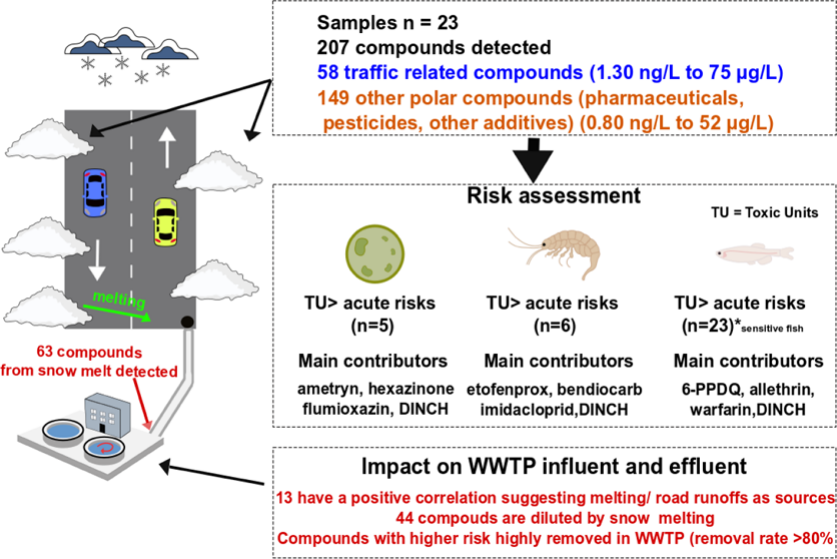

To assess the contamination and potential risk of snow melt with polar compounds, road and background snow was sampled during a melting event at 23 sites at the city of Leipzig and screened for 489 chemicals using liquid chromatography high-resolution mass spectrometry with target screening. Additionally, six 24 h composite samples were taken from the influent and effluent of the Leipzig wastewater treatment plant (WWTP) during the snow melt event. 207 compounds were at least detected once (concentrations between 0.80 ng/L and 75 μg/L). Consistent patterns of traffic-related compounds dominated the chemical profile (58 compounds in concentrations from 1.3 ng/L to 75 μg/L) and among them were 2-benzothiazole sulfonic acid and 1-cyclohexyl-3-phenylurea from tire wear and denatonium used as a bittern in vehicle fluids. Besides, the analysis unveiled the presence of the rubber additive 6-PPD and its transformation product *N*-(1.3-dimethylbutyl)-*N*′-phenyl-*p*-phenylenediamine quinone (6-PPDQ) at concentrations known to cause acute toxicity in sensitive fish species. The analysis also detected 149 other compounds such as food additives, pharmaceuticals, and pesticides. Several biocides were identified as major risk contributors, with a more site-specific occurrence, to acute toxic risks to algae (five samples) and invertebrates (six samples). Ametryn, flumioxazin, and 1,2-cyclohexane dicarboxylic acid diisononyl ester are the main compounds contributing to toxic risk for algae, while etofenprox and bendiocarb are found as the main contributors for crustacean risk. Correlations between concentrations in the WWTP influent and flow rate allowed us to discriminate compounds with snow melt and urban runoff as major sources from other compounds with other dominant sources. Removal rates in the WWTP showed that some traffic-related compounds were largely eliminated (removal rate higher than 80%) during wastewater treatment and among them was 6-PPDQ, while others persisted in the WWTP.

## Abstract 2

Road snow melt contains traffic-related chemicals and urban biocides at concentrations posing toxic risks to fish, crustaceans, and algae.

## Introduction

Urban stormwater and road runoff have been identified as a major source of toxic chemicals in surface waters either through direct discharges or in the case of combined sewer systems with municipal wastewater after treatment or without treatment in case of overflow.^1−3^ Next to biocides from facade runoff^4,5^ and multiple chemicals from atmospheric deposition,^6^ traffic-related emissions are assumed to be a major source of complex mixtures of pollutants in stormwater runoff contributing to the degradation of receiving waters.^7^ These emissions may originate from combustion of petrol, from tire and brake abrasion, leakage of different vehicle fluids, and abrasion of road surfaces.^3,8−13^ Recently, *N*-(1.3-dimethylbutyl)-*N*′-phenyl-*p*-phenylenediamine quinone (6-PPDQ), a transformation product of a globally used tire rubber antioxidant, was discovered as the cause for acute mortality of coho salmon (*Oncorhynchus kisutch*) in the United States.^14^ These results may also highlight the need to address traffic-related pollution beyond well-known traffic-related contaminants such as polycyclic aromatic hydrocarbons (PAHs)^6,15^ or metals.^16−19^ Rapidly advancing screening techniques applying liquid chromatography high-resolution mass spectrometry (LC-HRMS)^20^ have strongly enhanced the number of contaminants detected in wastewater,^21,22^ surface water,^23^ and urban runoff.^24^ Large-scale target, suspect, and non-target screening approaches further enhanced the opportunities to identify pollution patterns beyond well-known target chemicals.^25−27^

During winter time in the subpolar and temperate zone of the earth, snow may accumulate traffic-related chemical mixtures in the urban environment. With rising temperatures in spring, these compounds get mobile again, resulting in a subsequent short-term release of this pollution with snow melt runoff to sewer systems and surface waters.^28^ Thus, there is increasing awareness that snow may be a highly relevant matrix to monitor and understand urban and traffic-related environmental pollution as well as its impacts on aquatic ecosystems.^29^ Snow melt may contribute up to 60% to the chemical load in aquatic environments in cold regions^29,30^ at the end of winter.^29^ Previous investigations addressed, for example, total dissolved solids, chlorides originated from winter road maintenance,^31,32^ total suspended solids,^33^ tire-related compounds,^34,35^ and other traffic-related chemicals.^29^ However, a broad screening of contaminant mixtures in snow and snow melt together with the assessment of mixture toxic risks to aquatic organisms are still lacking. It is also unknown whether other chemicals used in urban life including pharmaceuticals and personal care products, biocides, additives to plastic and building materials, and others occur in snow and snow melt and how snow melt events translate to contamination patterns in wastewater treatment plant (WWTP) influents and effluents.

Thus, taking the city of Leipzig as an example, the aims of this study were (1) to screen urban snow for a large set of water contaminants including traffic-related and municipal-use chemicals using LC-HRMS, (2) to assess mixture toxic risks and identify the contribution of different sources, and (3) to study the impact of a snow melt event on the concentration profiles of contaminants in the influent and effluent of a WWTP.

## Methods

### Sampling Campaign and Sample Preparation

The sampling campaign was conducted in Leipzig (Germany) as a model urban area in February 2021. A period with temperatures >0 °C was followed by a cold snap on February 06/07, which included snowfall summing up to a cumulative height of about 20 cm (corresponding to about 25 mm of rain, see Supporting Information, Figure S1). The cold period lasted until February 16, when the temperatures increased to >0 °C and snowfall was succeeded by light rain. The temperature increase resulted in a complete melting of the snow until February 19. On the morning of February 17, 2021 between 8:00 and 12:00, twenty snow samples were collected directly on the sides of roads with different traffic intensities at a distance from 0.3 to 0.8 m to the roadway and will be referred to as road snow. These samples contained visible brownish-black particles and gritting material and were partly compacted and icy. In addition, three snow samples were taken in urban areas without traffic as a background reference (for details, see Supporting Information, Table S1). At each location, about 3–5 L of snow were collected in stainless steel containers and transported to the laboratory within 4 h. All samples were subsequently kept at −20 °C, and batches of 5–6 samples were melted overnight and filtered using a glass fiber filter (Whatman GF/F) to finally harvest 1 L of water. All samples were processed in this way within 4 days after sampling. The samples and two extraction blanks (1 L of LC/MS grade water) were enriched 1000-fold using solid phase extraction (SPE) (Method, see Supporting Information).^36^ The influent and effluent of the central WWTP Leipzig-Rosental (600,000 population equivalent) were collected for 24 h intervals during snow melting using on-site large volume solid phase extraction (LVSPE)^37,38^ of 20 L per sample from February 17th to February 22nd. Finally, all snow and WWTP sample extracts were re-dissolved in LC–MS grade MeOH with a concentration factor (CF) of 1000 and stored at −20 °C until LC-HRMS analysis.

### LC-HRMS Analysis

The samples were analyzed by LC–HRMS for 489 target compounds from different classes such as pesticides, biocides, pharmaceuticals, polymer and rubber additives, etc., derived from multiple literature sources (Supporting Information, Table S2). For analysis, 100 μL aliquots of the SPE extract were combined with 10 μL of an internal standard mixture containing 36 isotope-labeled compounds (1 μg/mL) (Supporting Information, Table S2), 30 μL of methanol (LC–MS grade), and 60 μL of water (LC–MS grade). The snow melt samples were analyzed at a final CF of 500, while the WWTP influent and effluent samples were analyzed at a final CF of 5 and 50, respectively. Extract aliquots of 5 μL were injected into the LC system (Thermo Ultimate 3000 LC), and separate runs were conducted with electrospray ionization in positive and negative ion modes. For HRMS detection using a QExactive Plus (Thermo Scientific), a full scan acquisition (*m*/*z* 100–1500, nominal resolving power 70,000) was combined with data-independent acquisition in different *m*/*z* windows (nominal resolving power 35,000). Instrumental blanks (methanol/water 70:30) were analyzed in the same batch (for details, see the Supporting Information).

Target compounds were quantified in both SPE and LVSPE extracts by our in-house method using matrix-matched calibration in filtered water from a pristine reference stream (Wormsgraben) located in the upper Harz Mountains. 1 L aliquots were spiked with mixtures of all target compounds (Supporting Information, Table S2) at 13 levels ranging from 0.1 to 5000 ng/L. These calibration standards were processed the same way using 200 mg of HR-X as the SPE samples.

### Data Processing

Chemicals were identified and quantified with a workflow involving ProteoWizard 3.0, MZmine 2.38, and the MZquant R-package.^21^ Briefly, Thermo raw files obtained from the LC-HRMS were converted to the .mzML format using ProteoWizard^39^ (3.0). Peak picking, deconvolution, alignment, gap filing, and peak annotation were performed using MZmine 2.38 as described in Beckers et al., 2020.^26^ Annotated files were exported as csv, and compounds were quantified with the in-house R package MZquant^40^ (https://git.ufz.de/wana_public/mzquant/-/releases/0.7.22) as detailed in the Supporting Information, Section S2. The identity of the detected compounds was checked using one or two diagnostic MS^2^ fragment ions using the vendor software TraceFinder (version 4.1, Thermo Scientific). Compounds, which could not be reliably quantified using MZmine/MZquant (e.g., poor peak integration), were fully quantified using the TraceFinder software. Method detection limits (MDLs) were estimated from replicate injections of the calibration standards based on the US EPA method.^41^ Detailed information concerning MDLs can be found in Supporting Information, Table S2.

The detected compounds were then aggregated according to their usage and probable sources (human consumption including pharmaceuticals, food ingredients, personal care products, dyes, etc; pesticides and biocides; traffic-related compounds; and other chemicals of interest). Compounds with multiple uses including traffic-related ones were grouped into the traffic-related compounds category as the most probable source.

Data were analyzed and visualized in R 4.0.4^42^ using ggplot2^43^ and corrplot^44^ packages. The city map from Leipzig was downloaded from geoportal.de (last accessed March 24, 2022). The sampling sites were located according to their GPS coordinates. The figure’s layout was obtained using the design software Inkscape V.0.9.4.

### Mixture Toxic Risk Assessment

Mixture toxic risks of chemicals in snow samples were evaluated using a toxic unit (TU) approach^45,46^ defined as the ratio of the measured environmental concentration (MEC) to the acute 50% effect concentration (EC_50_) values available per biological quality element (BQE) on algae, crustacean, and fish for each compound *I* (Equation 1).
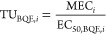
1

In agreement with previous assessment of wastewater samples,^22^ effect data (EC_50,BQE,*i*_) were used in the following order: (1) experimental data (5^th^ percentile of all EC_50_ values available per BQE) retrieved from the US-EPA ECOTOX database (https://cfpub.epa.gov/ecotox, 15.6.2021) or, if no experimental data were available, (2) predicted EC_50_ values using the ECOSAR type baseline toxicity model for the BQEs fish, daphnia, and green algae in Chemprop 6.7.1 (UFZ Department of Ecological Chemistry, 2019). The detailed database is available on Zenodo (https://zenodo.org/record/6137082#.YvF2G3a-g2w). Mixture risks were calculated by the summation of all TU_*i*_ per organism group of the detected target compounds, yielding the TUsum (Equation 2).
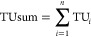
2

This method is based on the assumption of concentration addition.^47^ All samples were evaluated and prioritized according to the exceedance of chronic TUsum risk thresholds suggested by Malaj et al., 2014^45^ and applied by Kandie et al., 2020:^48^ for algae (TUsum = 0.02), daphnia, (TUsum = 0.001), and fish (TUsum = 0.01).

## Results and Discussion

### Chemical Profiles of Urban Snow Melt Samples

**Figure 1:**
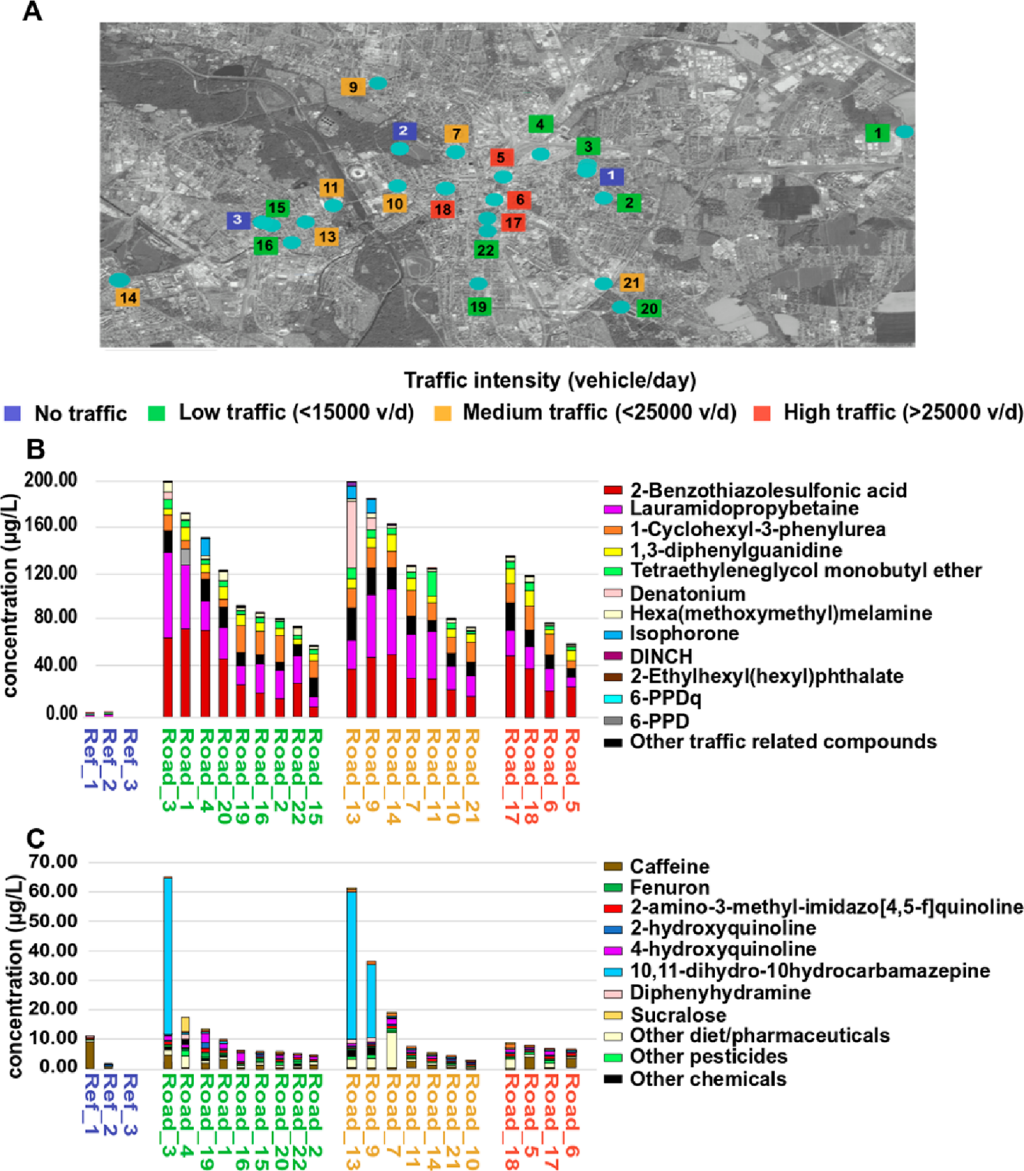
Spatial distribution of polar compounds in urban areas with high-density traffic. (A) Locations of snow samples collected in the city of Leipzig. (B) Distribution of traffic-related compounds found with concentrations higher than the detection limits. (C) Distribution of other polar compounds found with concentrations higher than the detection limits.

Snow melt samples were analyzed from 23 locations considering different traffic levels (Figure 1A). The chemical analysis unveiled that 207 compounds were at least detected once in any of these sample concentrations between 0.80 ng/L and 75 μg/L (Supporting Information, Table S3) including 58 traffic-related compounds (concentrations from 1.30 ng/L to 75 μg/L) (Figure 1B) and many other compounds such as pharmaceuticals, pesticides, food additives, and others (149 compounds in concentrations from 0.80 ng/L to 52 μg/L) (Figure 1C). In detail, 128 compounds could be quantified in only one sample, showing a highly specific contamination. Snow melt samples collected at road sides contained between 80 and 128 compounds, while snow melt samples collected in backyard areas contained between 30 and 41 compounds. The details are given in Supporting Information, Table S3.

The summed concentrations of traffic-related compounds in road snow varied between 59 and 200 μg/L (average 118 ± 44 μg/L) (Figure 1B). Backyard sample concentrations were consistently lower with summed concentrations up to 4.5 μg/L. Traffic-related mixtures exhibited a quite consistent pattern with 2-benzothiazole sulfonic acid (*n* = 20 from 8 to 75 μg/L), lauramidopropyl betaine (*n* = 20 from 8 to 72 μg/L), 1-cyclohexyl-3-phenylurea (*n* = 20 from 6 ng/L to 22 μg/L), 1.3-diphenyl guanidine (*n* = 20 from 3 to 14 μg/L), tetraethyleneglycol monobutyl ether (*n* = 20 from 2 to 20 μg/L), and 3-cyclohexyl-1,1-dimethylurea (*n* = 20 from 40 ng/L to 4 μg/L) as the top six compounds (based on the concentration) found in the snow melt samples. No dependency of total concentrations and chemical patterns on traffic intensity could be observed (details are given in Supporting Information, Table S3). Also, the tire wear compounds and antioxidant 6-PPD (*N*-[1.3-dimethylbutyl]-*N*′*-*phenyl-*p*-phenylenediamine) and its transformation product (6-PPDQ) were detected in the road snow samples at concentrations of 0.21 ± 0.23 μg/L (*n* = 13 from 65 to 783 ng/L) and 3.3 ± 2.0 μg/L (*n* = 20 from 110 to 428 ng/L), respectively. The results show that these tire wear compounds, which are known to be harmful to aquatic organisms^14,24,49,50^ and thus deserve our specific attention, are accumulated in the snow.

Benzothiazole derivatives are common products of vulcanizing^51^ and well known as traffic-related pollutants as they show high detection frequencies in road runoff.^24^ In agreement with previous studies, benzothiazole sulfonic acid, being a stable and hydrophilic oxidation product of different derivatives, was the most prominent substance in road runoff within this compound group with similar concentrations of 40 to 50 μg/L.^24,52^ For the rubber additive and polymerization catalyst 1-cyclohexyl-3-phenylurea, we found 40-fold higher concentrations compared to previous studies on road runoff,^53,54^ while 1,3-diphenylguanidine, a vulcanization accelerator, was detected in concentrations about five-fold lower than in road runoff samples analyzed previously.^24^ Concentrations of the anti-oxidant 6-PPD and its transformation product 6-PPDQ were in agreement with concentrations reported previously in road runoff.^55^ In addition to these tire wear compounds, other traffic-related compounds detected in this study have their origin likely in vehicle fuels and fluids. This holds, for example, true for 3-cyclohexyl-1,1-dimethylurea, which is used in traction drive oils, for tetraethyleneglycol monobutyl ether, which is a widely used vehicle antifreeze,^56^ and tetraglyme, which may be used in lithium ion batteries. More surprisingly, the surfactant lauramidopropyl betaine and the bittering agent denatonium were also found at high concentrations in the samples. Lauramidopropyl betaine is used in personal care products, pet shampoo, or household detergents^57,58^ and can also be found in car cleaning agents, which might explain its presence. Denatonium is added to products containing alcohols or ethyleneglycols such as antifreeze and coolants in order to discourage consumption and was detected in concentrations well in agreement with a previous study on stormwater.^24^

Non-traffic compounds are found with cumulative concentrations from 3.5 to 65 μg/L (average 15 ± 17 μg/L) (Figure 1C), underlining that these compounds are not neglectable. The maximum cumulative concentration of non-traffic-related chemicals in backyard samples was much lower, amounting to 3 μg/L (2.5 ± 0.5 μg/L). Concerning the background snow, the presence of nitrophenols as major compounds contributing to the chemical profile could be noticed. Nitrophenols should mainly come from atmospheric deposition. The top five compounds, based on their concentrations in the road snow melt samples, were 10,11-dihydro-10 hydroxycarbamazepine (*n* = 20 from 80 ng/L to 52 μg/L), caffeine (*n* = 20 from 304 ng/L to 5 μg/L), 2-hydroxyquinoline (*n* = 20 from 219 ng/L to 2 μg/L), 2-amino-3-methyl-imidazo [4.5-f] quinoline (*n* = 20 from 385 ng/L to 1 μg/L), related to human consumption and the herbicide fenuron (also used as a rubber additive) (*n* = 19 from 297 ng/L to 1 μg/L). Besides, there were five other compounds with an average concentration higher than 50 ng/L including cotinine, a nicotine metabolite (*n* = 20 from 45 to 559 ng/L), hypertension pharmaceutical metoprolol acid (*n* = 20 from 53 to 269 ng/L), enalapril (*n* = 9 from 4 to 732 ng/L), mepiquat, a plant growth inhibitor (*n* = 20 from 86 to 254 ng/L), and the local anesthetic tetracaine, which is used, for example, in eye drops (*n* = 17 from 2 to 748 ng/L). Most of the compounds detected may stem from improper disposal of coffee (cups) and cigarettes, but also from urination. Cotinine and caffeine were found in snow melt in a similar range to that in stormwater.^24^ Similar pathways may be assumed for compounds related to food or food packaging and pharmaceutical compounds that were found in the snow melt in a large number of samples. Some of the pharmaceuticals may also be related to pet urination as they are known to be used in some veterinary drugs (metoprolol and carbamazepine). These findings are in line with the literature mentioning their detection at low concentrations in road runoff^24^ (tetracaine and carbamazepine as the parent compound of 10,11-dihydro-10-hydroxycarbamazepine).

Finally, 64 pesticides and biocides were found, which is almost one-third of the compounds detected. 2-Aminobenzimidazole, 2-octyl-4-isothiazolin-3-one, allethrin, bendiocarb, carbendazim, DEET, diuron, fenoxycarb, icaridin, terbutryn, and warfarin among them are used as biocides. While most of them occurred at relatively low concentrations below 50 ng/L, there were some pesticides that occurred at higher concentrations. This included fenuron, the insecticide bendiocarb (*n* = 10 from 4 to 408 ng/L), the herbicide simazine (prohibited in Germany since 1991) (*n* = 17 from 2 to 820 ng/L), the insecticide allethrin (*n* = 20 from 63 to 186 ng/L), and the herbicide diflufenican (*n* = 12 from 37 to 189 ng/L). The low concentrations of various pesticides are in line with a previous study underlining the large variety of compounds with low concentrations.^24^ The biocides and pesticides may stem from roadside vegetation control^59^ and from leaching off building facades and roofs.^24^ The pesticide concentration, especially for herbicides, might be lower in snow melt than other runoff examples as their use is limited in winter.

### Mixture Risk Assessment and Risk Drivers in Snow Melt Samples as a Proxy of Road Runoffs

**Figure 2:**
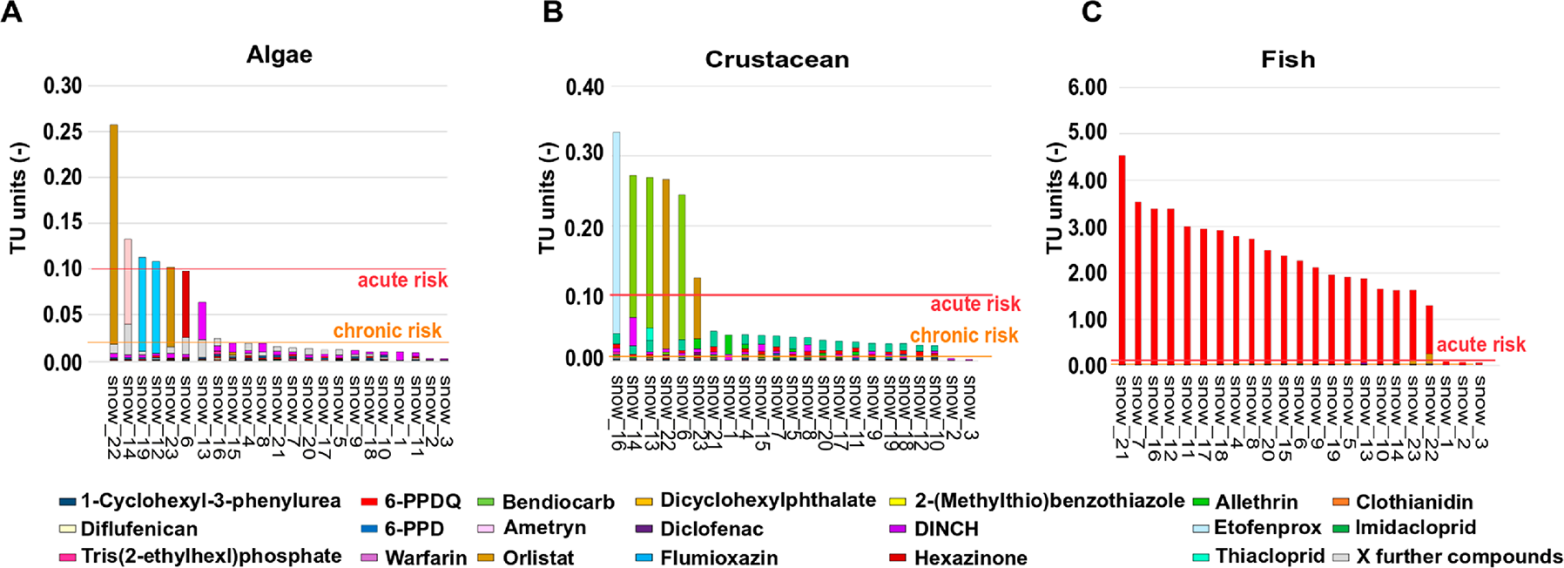
Risk characterization of the different snow melt samples for (A) algae, (B) fish, and (C) crustaceans. The bar plots represent the sum of TU of the different compounds at each site. Compounds with the largest contribution to the TU and compounds with higher contributions are named in this figure, all the other compounds’ TU with minor contributions are summarized in the further compounds.

To evaluate the potential risks of the different snow melt samples, TUs were calculated for each compound and the resulting mixture toxicity (cumulative TU) at each site sampled. Figure 2 shows the cumulative TU found in each sample for three aquatic organism groups: algae (Figure 2A), crustacean (Figure 2B), and fish (Figure 2C). Assuming chronic toxicity thresholds of 0.02 TU for algae, 0.001 TU for crustaceans, and 0.01 TU for fish,^45^ eight samples exceeded the chronic risk threshold for algae, with five of them also exceeding the acute risk threshold. For crustaceans, all traffic-related samples exceeded the chronic risk threshold with six samples exceeding also the acute risk threshold, while all snow melt samples in traffic areas exceeded both chronic and acute risk thresholds for fish (considering sensitive fish such as coho salmon or rainbow and brook trout^60^). Considering the ecological and economical importance of trout and other Salmonidae as carnivory and food fish in Germany, it seems interesting to perform a specific study focused on these sensitive fish.

The risk for the algae is driven by site-specific chemicals including the obesity drug orlistat detected at two sites with 0.08 and 0.23 TU; the herbicides ametryn (*n* = 1, TU = 0.09), hexazinone (*n* = 1, TU = 0.07), and flumioxazin (*n* = 2, TU = 0.01); and the plasticizer 1,2-cyclohexane dicarboxylic acid diisononyl ester (DINCH) (*n* = 1, TU = 0.04) (Figure 2A). Ametryn and hexazinone where among the top 30 risk drivers for algae in municipal wastewater, supporting the urban-use-related risks of these chemicals,^22^ although the use in Germany was banned in 1994 and 2001, respectively. Large log *K*_ow_ tend to overestimate the risk in linear models such as ECOSAR. The predicted EC_50_ values exceeded the threshold of 10^5^ × log_10_*S*_*i*_ (where log_10_*S*_*i*_ is the logarithm on base 10 of the solubility of the compound *i* in mg/L). Thus, the predicted EC_50_ value was replaced by the water solubility, as the EC value could not be greater than the solubility of the compound. The half log unit tolerance addresses issues of uncertainties in the log *S* estimation. In the case of compounds with low (predicted) solubility, this approach may cause an increase of the predicted toxicity. The compounds with the highest contribution for the toxic profile were detected in low concentrations but higher than the MDL (two-fold), restricting analytical uncertainties. The large contributions of DINCH and orlistat to mixture risks to algae are not based on experimental but solely predicted EC_50_ values using ECOSAR models and are related to their high predicted log *K*_ow_ values. Thus, these values bear a substantial uncertainty. For both chemicals, experimental toxicity data are required to confirm the risk.

The main compounds contributing to the risk to crustaceans (Figure 2B) were the insecticides etofenprox at one site (*n* = 1, TU = 0.27) and bendiocarb at three sites with about 0.2 TUs. The latter is a typical component of antiparasitic dog collars, explaining the occurrence in road snow. At the two sites contaminated with the drug orlistat, this compound substantially contributed to the mixture risk (*n* = 2, TU between 0.23 and 0.08) based on the estimated toxicity. Further risk contributors include DINCH (*n* = 2, TU between 0.01 and 0.04), the neonicotinoid insecticides thiacloprid (*n* = 1, TU = 0.01) and imidacloprid (*n* = 2, TU between 0.01 and 0.02), and the pyrethroid insecticide allethrin, which is also used as an insect repellent (*n* = 14, TU between 0.01 and 0.02). Both neonicotinoids and pyrethroids are well known for their risk to aquatic invertebrates.^61,62^

The rubber-additive 6-PPDQ mainly explained the exceedance of risk thresholds for fish if sensitive species such as coho salmon are considered (*n* = 23, TU between 0.05 and 5.4). Similar to algae and crustaceans, orlistat contributes at the two contaminated sites also to the risk to fish (*n* = 2, TU between 0.08 and 0.23) (Figure 2C). Other fish risk contributors included the rodenticide and pharmaceutical warfarin, which might originate from rat control (*n* = 1, TU = 0.01), DINCH (*n* = 2, TU between 0.01 and 0.04), and allethrin (*n* = 13, TU between 0.01 and 0.02). The concentrations of 6-PPDQ exceeded the EC_50_ value of coho salmon of 0.000095 mg/L (24 h juvenile coho salmon exposure)^14^ in all snow melt samples. First results showed that 6-PPDQ toxicity is probably dependent on the fish or aquatic organisms species considered,^63^ but this compound has been tested only for a small number of species so far. However, it cannot be excluded that snow melt events and road runoffs could impact sensitive species and their communities in European rivers, especially Salmonidae like *Salmo trutta*, *Salvelinus*, and *Salmo salar*. For species insensitive to 6-PPDQ, the toxic risk is mainly related to orlistat, DINCH, allethrin, and warfarin. Allethrin contributed to chronic risk to fish in more than half of the samples due to its great toxicity to fish.^64^ All details concerning the individual TUs are found in Supporting Information, Tables S4–S7.

### Impact of a Snow Melt Event on Concentrations in WWTP Influents and Effluents

**Figure 3:**
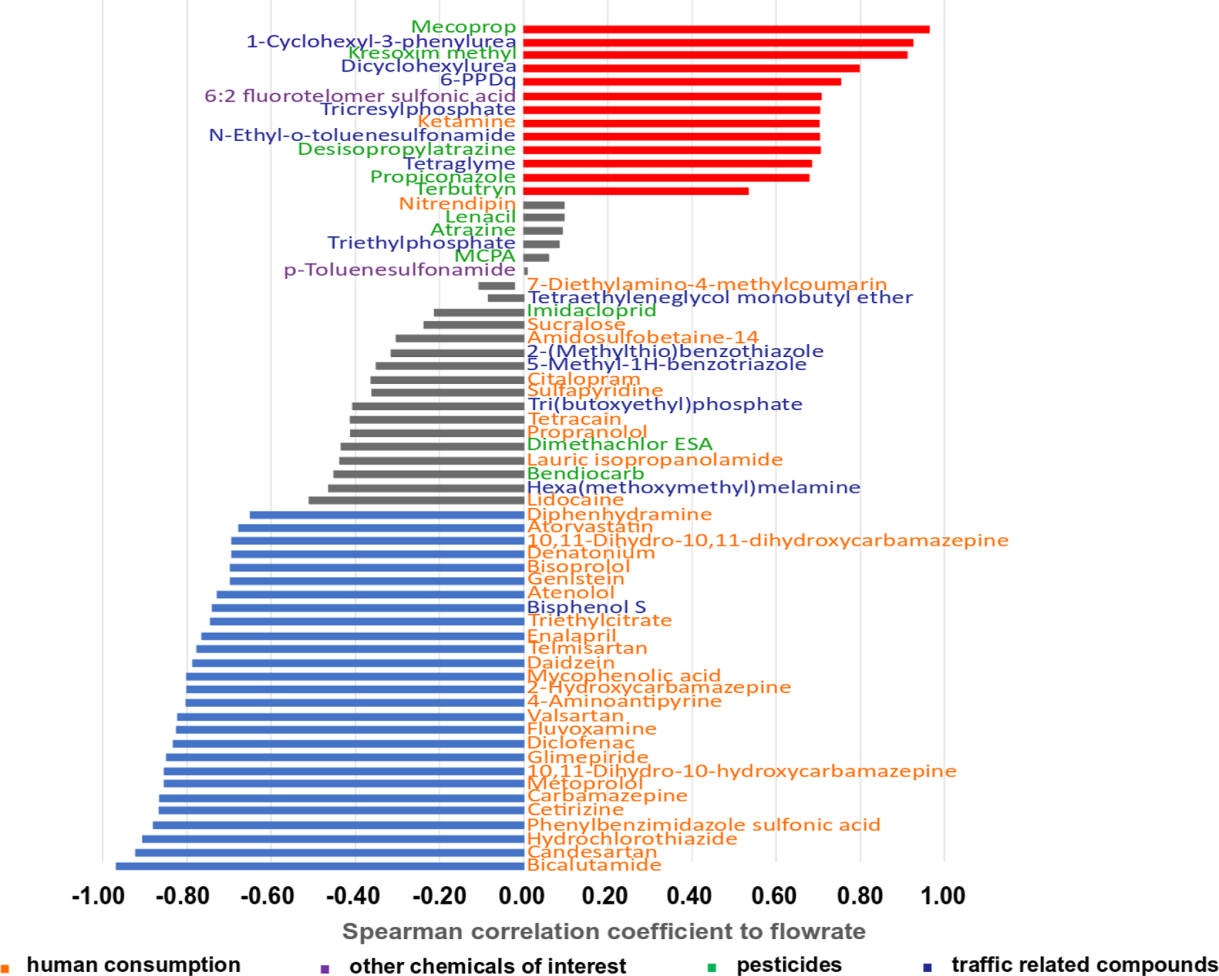
Spearman correlation coefficient between compound concentrations and the WWTP influent flow rate. The bar plots in red indicate a positive correlation and the ones in blue a negative correlation.

In total, 63 out of 207 compounds found in the snow melt samples (Supporting Information, Table S8) were also detected in the influent to the WWTP. To determine which of these compounds are mainly related to the runoff due to snow melt, the compound concentrations in the WWTP influent were correlated to the inlet flow rate measured in the WWTP using the Spearman correlation (Figure 3). This approach assumes that concentrations increase with discharge only if they actually originate in the runoff (strong positive correlation, red bars), while runoff dilutes compounds with other sources (household wastewater) (strong negative correlation, blue bars). Examples are shown in Supporting Information, Figure S3A–C. Correlations with a Spearman correlation coefficient below 0.5 (absolute value) were assumed to indicate more complex discharge patterns from different sources.

A positive correlation (coefficient above 0.5) was observed for 13 chemicals. Among these 13 compounds, six are clearly traffic-related including 1-cyclohexyl-3-phenylurea, diclohexylurea, 6-PPDQ, tricresylphosphate, *N*-ethyl-*o*-toluenesufonamide, and tetraglyme, while five chemicals are used as herbicides and fungicides in urban environments including mecoprop, kresoxim methyl, propiconazole, desisopropylatrazine (TP of legacy agricultural herbicides), and terbutryn. The high contributions from traffic-related compounds and urban pesticides are in line with previous findings.^24^ For example, terbutryn is a common biocide used as a film preservative in facade paints^65^ and may be washed out during rain events or snow melting. The same holds true for mecoprop, which is used in bitumen membranes against root penetration in roofs^66,67^ and propiconazole which is applied for wood preservation.^68^ Interestingly, also the human and veterinary pharmaceutical ketamine and the polyfluorinated acid 6:2 fluorotelomer sulfonic acid were positively correlated to the wastewater flow during snow melt. 6:2 fluorotelomer sulfonic acid as a per andpolyfluoroalkyl substance (widely use as anticorrosion compounds) could accumulate in road dust^69,70^ and be released in sewer waters during snow melting.

In total, 44 compounds exhibited a negative correlation coefficient, with 27 of them showing a correlation coefficient greater than 0.5 (Figure 3). This indicates that these compounds originated from other dominants sources as their concentrations got diluted by snow melt-induced urban runoff. These compounds include primarily pharmaceuticals stemming from domestic or health-care-related wastewater sources and are diluted during snow melt.

**Figure 4:**
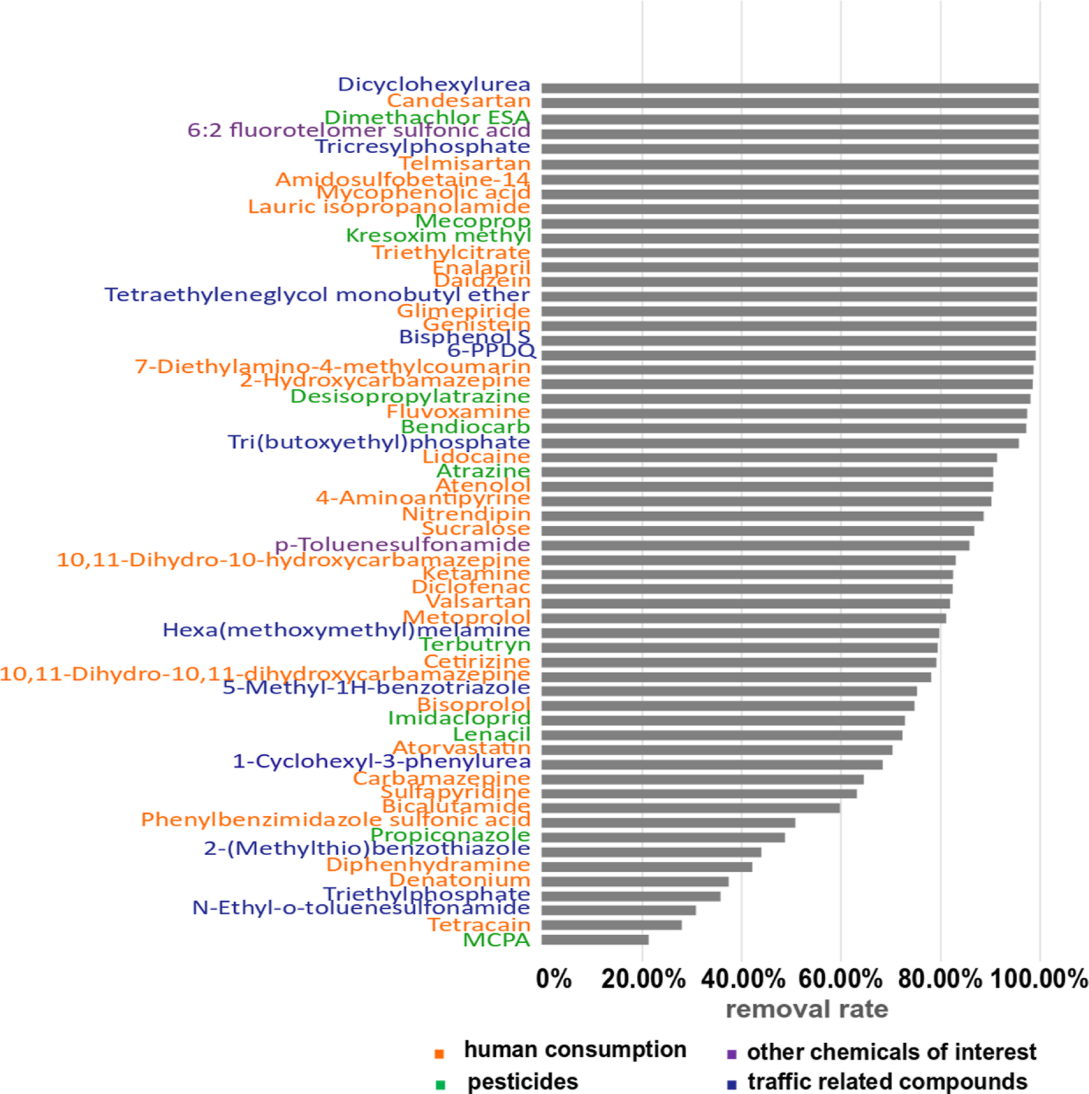
Removal rate of compounds found in both the WWTP influent and snow melt. The removal rate from cumulative loads of the total snow melting period (7 days) to minimize the effect of highly fluctuating flow rates and hydraulic residence times.

To better understand the potential impact of snow melt-related compounds on the WWTP effluent and hence the quality of the water discharged into adjacent waterbodies, effluent concentrations were determined for those chemicals detected in snow melt and the WWTP influent. Influent and effluent loads were estimated for the snow melt period under consideration (from February 17th to February 23th) and used to estimate a removal rate for all compounds found both in snow and in the influent (Figure 4 and Supporting Information, Tables S9–S11). In total, 13 compounds detected in the influent fell below the MDL in the effluent; among them were five out of the 13 compounds strongly related to snow melt-induced runoff. In addition to these 13 compounds, 27 compounds (three out of the 13 strongly related to snow melt) were identified in the effluent but exhibited a removal rate greater than 80%. Among these compounds, three were strongly related to snow melt. This group of compounds includes 6-PPDQ as the dominant toxicity driver for sensitive fish with a concentration in the WWTP effluent of 5 ng/L (below the toxicity threshold) and a removal rate of 99%. Thirteen compounds exhibited a removal rate between 50 and 80%. Eight compounds (MCPA, tetracain, *N*-ethyl-*o*-toluenesulfonamide, triethylphosphate, denatonium, diphenhydramine, 2-methylthiobenzothiazole, and propiconazole) were found with a lower removal rate. Finally, four compounds (hydrochlorothiazide, citalopram, propranolol, and tetraglyme) given in Supporting Information, Table S11 showed a negative removal rate. The transformation of micropollutants (i.e., deconjugation) as well as the matrix effect (less organic matter in the effluent)^71^ could partly explain the negative removal rate observed in this case. Thus, among the compounds closely related to snow melt, particularly propiconazole, *N*-ethyl-*o*-toluenesulfonamide, and tetraglyme are hardly retained in the WWTP. For the other compounds including 6-PPDQ and other risk drivers, highly efficient removal processes might protect the aquatic environment from excessive toxicity; however, if an overflow of the WWTP occurs, detrimental effects (especially for fish) are likely.

The study provides an overview on the contamination of road snow with polar organic chemicals, which extends beyond well-known contaminants such as metals and PAHs. Highly consistent patterns of traffic-related chemicals were found in road snow without any clear dependence on traffic intensity, but they could be clearly discriminated from backyard snow samples as a reference. The traffic-related mixtures were complemented with rather site-specific compounds including pesticides, pharmaceuticals, and food additives. The tire-wear-related oxidation product of 6-PPD, namely, 6-PPDQ, occurred in all road snow samples. The observed concentrations suggest an acute toxic risk to sensitive fish (e.g., coho salmon or rainbow trout). In contrast, mostly non-traffic-related contaminants were the drivers of acute toxic risks for algae and crustaceans in snow melt. Therefore, other sources need to be taken into account. Urban-use biocides and the insecticide bendiocarb applied in dog collars are potential risk drivers beyond individual sites only. Correlations between the WWTP influent flow rate and chemical concentrations allowed us to discriminate compounds with urban runoff as the dominant source from compounds identified in road snow but with other dominant sources. The concentrations of compounds having urban runoff as the dominant source increase in influent samples, while the others compounds are diluted by snow melt runoff (e.g., pharmaceuticals and personal care products).

Many compounds emitted with snow melt are not well removed in the investigated WWTP and contribute to toxic risks in receiving waters. Besides, risk drivers such as 6-PPDQ should also be monitored even if their concentrations are strongly reduced. Indeed, snow melt period or heavy rain events could generate sewer overflow and high discharge periods and create a source of toxic contamination in surface waters.

## Supporting Information Available

Details on sampling points of snow samples; target list and isotope compounds; concentration of chemical compounds; toxic units of individual compounds for algae, crustacean, and fish; EC_50_ values; concentration in the WWTP influent; concentrations in the WWTP effluent; discharges of the WWTP; and compounds’ removal rate in the WWTP (XLSX)
Additional information on the sampling campaign and sample preparation, chemical analysis, chemical profiles of snow melt samples, risk assessment, and impacts on the WWTP influent and effluent (PDF)
