# Supplementary material for: Contamination Pattern and Risk Assessment of Polar Compounds in Snow Melt: An Integrative Proxy of Road Runoffs
Source: Environ Sci Technol. 2023 Mar 2;57(10):4143–52. doi: 10.1021/acs.est.2c05784 (PMC10018729; doi:10.1021/acs.est.2c05784)
Supplement: Supplementary file 2 — es2c05784_si_002.pdf [file es2c05784_si_002.pdf]

Supporting Information

Contamination pattern and risk assessment of polar compounds in snow melt: an integrative proxy of road runoffs

Loïc Maurer[a*#], Eric Carmona[a], Oliver Machate[a], Tobias Schulze[a], Martin Krauss[a], Werner Brack[a,b]

[a] UFZ—Helmholtz Centre for Environmental Research, Department of Effect-Directed Analysis, Permoserstr. 15, 04318 Leipzig, Germany.

[b] Institute of Ecology, Evolution and Diversity—Goethe University,Max-von-Laue-Str. 13, 60438 Frankfurt am Main, Germany.

*Email contact loic.maurer@engees.unistra.fr

Present Addresses:

Université de Strasbourg, CNRS, ENGEES, ICube UMR 7357, F-67000 Strasbourg, France

Keywords: Chemicals of Emerging Concern (CECs), Liquid Chromatography High Resolution Mass Spectrometry (LC-HRMS), snow melt, urban road runoffs, risk assessment, tire wear compounds, pesticides, pattern analysis

Loic Maurer: 0000-0002-7248-4804
Eric Carmona: 0000-0002-1834-8342
Oliver Machate: 0000-0002-4247-8358
Martin Krauss: 0000-0002-0362-4244
Tobias Schulze: 0000-0002-9744-8914
Werner Brack: 0000-0001-9269-6524

9 Pages

3 Figures

11 Tables

Table of contents

Section S1: Additional information on sampling campaign and sample preparation 2
Section S2: Additional information on chemical analysis...2
Section S3: Additional information on chemical profile of snow melt samples 4
Section S4: Additional information on risk assessment 4
Section S5: Additional information on impacts on WWTP influent and effluent..........................4

Section S1: Additional information on sampling campaign and sample preparation

Sampling campaign

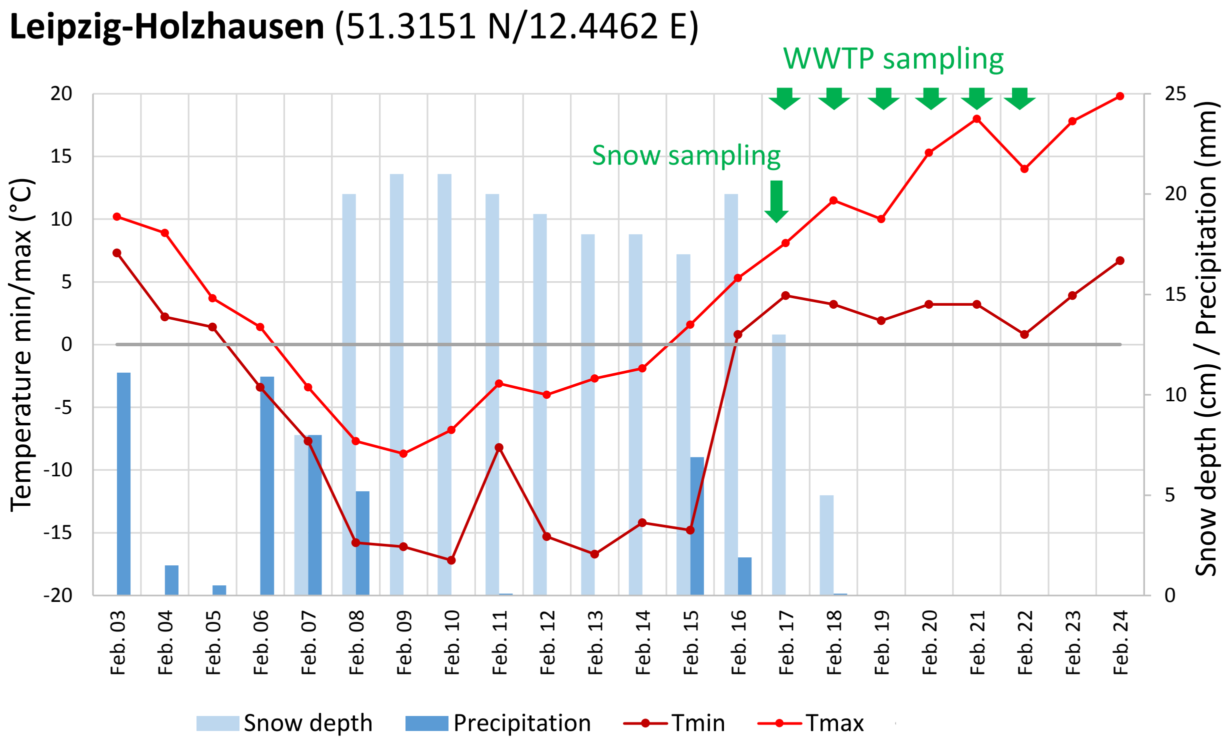

Data source: Deutscher Wetterdienst, Climate Data Center (CDC), https://cdc.dwd.de/portal/, accessed 03 Nov 2022

Figure S1: Minimum and maximum daily temperatures (at 2 m above soil height), daily precipitation and snow cover depth before and during the sampling campaign in February 2021 as recorded at the meteorological station Leipzig-Holzhausen.

The sampling sites are listed in Table S1

Table S1: Sampling points of snow samples in Leipzig city

Solid-phase extraction of water samples

Suspended solids were removed with Whatman GF/F glass microfiber filters with a pore size of 0.7 µm fusing a vacuum pump. Filtered samples where extracted with Chromabond HR-X cartridges (6 mL, 200 mg sorbent, Macherey-Nagel, Düren, Germany). Methanol and ethyl acetate (both LC/MS grade) and, LC/MS-grade water was obtained from Fisher Scientific (Schwerte, Germany) and formic acid (98 – 100 %; for analysis) was ordered from Merck (Darmstadt, Germany). The extraction was conducted using a SPE-03 device (Promochrom, Richmond, BC, Canada). Cartridges were conditions with 5 mL of ethyl acetate, followed by 5 mL of methanol and 5 mL of LC-MS grade water and 1 L of snow melt sample waspassed through the cartridge with a flow rate of 10 mL/min. Subsequently, cartridges were dried in a nitrogen stream for 30 minutes and dry cartridges were eluted with 5 mL of ethyl acetate, followed by 5 mL of methanol, 5 mL of methanol containing 1% of formic acid and 5 mL of methanol containing 2% of 7 N ammonia in methanol. Extracts were evaporated under a nitrogen flow to nearly dryness and adjusted with methanol to obtain a concentration factor (CF) of 1000 (i.e., a 1 L water sample yields 1 mL of extract).

Large volume solid-phase extraction of wastewater samples

One LVSPE device (as described in Schulze et al., 2017[1], Välitalo et al., 2017[2]) was deployed each at the influent and the effluent of the Leipzig-Rosental WWTP from Feb 17, 2021 9:00 to Feb 23, 2021, 9:00. Six time-proportional 24-hour composite samples for both, influent and effluent were taken. During extraction, samples were filtered using a Sartopure GF + MidiCap, 0.65 µm deep filter (Sartorius) and extracted using cartridges filled with 10 g of Chromabond HR-X (Macherey-Nagel). The latter were conditioned with 200 mL of ethyl acetate (LC-MS grade) followed by 200 mL of methanol (LC-MS grade) and 100 mL of LC-MS grade in the laboratory before sampling. After extraction, the cartridges were dried in a nitrogen stream in the laboratory and afterwards freeze-dried to remove residual water. The cartridges were eluted with 100 mL of ethyl acetate, 100 mL of methanol, 100 mL of methanol containing 1 vol% of formic acid (98–100%, p.a., Merck), and 100 mL methanol with 2 vol% of 7 N ammonia in methanol (Sigma- Aldrich). One processing blanks were prepared by extracting 5 L of LC-MS grade water by LVSPE and transporting/processing them together with the sampling cartridges. All extracts were blown down to about 1 mL, re-dissolved in LC-MS grade methanol at a CF of 1000 and stored at –20 ◦C until further analysis.

Section S2: Additional information on chemical analysis and data processing

Table S2: Target list and internal standard used for the chemical analysis

LC-HRMS analysis

Compounds were analyzed using a Thermo Ultimate 3000 LC coupled via a heated electrospray ion source to a Thermo QExactive Plus MS. Prior to injection, 100 µL aliquots were transferred into 2 mL autosampler vials after filtration and 30 µL of methanol, 60 µL of water (LC-MS grade) and 10 µL of an internal standard solution containing 36 isotope-labelled compounds in methanol were added. Calibration standards were prepared in surface water from a stream (Wormsgraben) with no anthropogenic impact located in the upper Harz mountains at levels of 1, 2, 5, 10, 20, 50, 100, 200, 500, 1000, 2000 and 5000 ng/L in the same way as the samples.

Compounds were separated using a Kinetex C18 EVO column (50 × 2.1 mm, 2.6 µm particle size, Phenomenex, pre-column 4 x 2.1 mm and in-line filter 0.2 µm) and a gradient elution with 0.1% of formic acid and methanol containing 0.1% of formic acid at a flow rate of 300 µL/min. After 1 min of 5% B, the fraction of B increased to 100% within 12 min and 100% B were kept for 11 min. The eluent flow was diverted to waste and the column was rinsed for 2 min using a mixture of isopropanol and acetone 50:50 / eluent B / eluent A (85% / 10% / 5%) to remove hydrophobic matrix constituents from the column. Subsequently, the column was re-equilibrated to initial conditions for 5.7 min. The column temperature was set to 40°C. Separate runs were conducted with electrospray ionization in positive and negative ion mode in a full scan experiment (100-1500 m/z) at a nominal resolving power of 70,000 (referenced to m/z 200) with twelve different isolation windows. These isolation windows were 50 mu (i.e., m/z ranges 97-147, 144-194, 191-241, 238-288, 285-335, 332-382, 379-429, 426-476) and 260 mu (i.e., m/z ranges 473-733, 729-989, 985-1245, 1241-1501), respectively. Mass calibration of the MS instrument was done before each sequence by direct infusion of the calibration solution recommended by Thermo.

After conversion of the Thermo raw files to the mzML format using ProteoWizard,[3] peak detection was done in MZmine 2.38[4] using a 7 ppm mass window for extracting ion chromatograms from the full scan data and annotation of the target compounds in the peak list.
Peak picking, deconvolution, alignment, gap filing and peak annotation were performed using MZmine 2.38 as described in Beckers et al., 2020[5].
For quantification of the target compounds, annotated peak lists were exported as .csv files and the R package MZquant (https://git.ufz.de/wana_public/mzquant/-/releases/0.7.22) was used. It included first an automated blank peak removal, which removed all peaks with intensities < mean intensity + 2 standard deviations of all instrumental and sample processing blanks. For the internal standard calibration, for each target compound the closest eluting internal standard was assigned to correct for matrix effect differences among samples and calibration standards. The calibration functions were trimmed to the response ratio range observed in samples to allow for a better fitting of the calibration curve in the relevant concentration range. A quadratic regression was fitted to the calibration points using a generalized additive model from the R package mgcv. The quality of fit was confirmed visually and based on R^2 values as well as residuals

Section S3: Additional information on chemical profile of snow melt samples

Table S3: Concentration of chemical compounds found in the snow melt samples

Section S4: Additional information on risk assessment

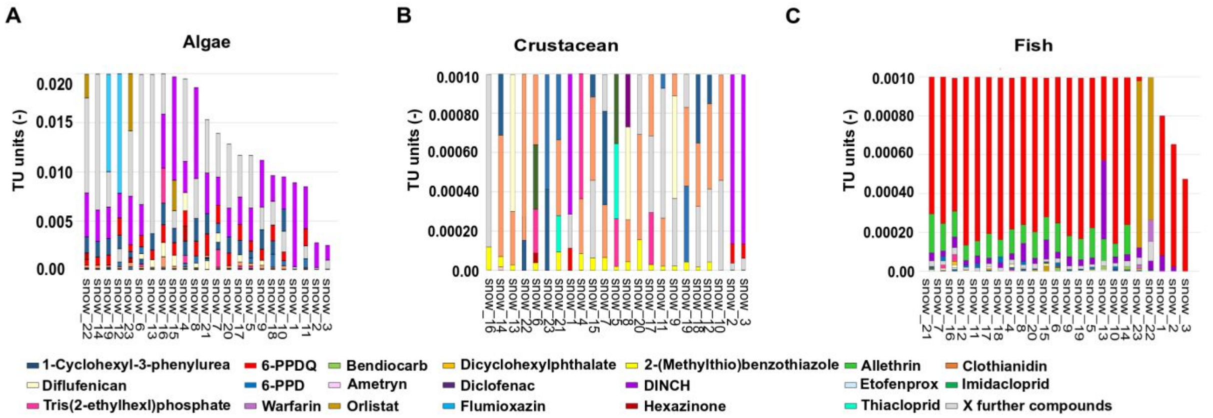

Figure S1 Risk characterisation of the different snow melt samples for A algae, B fish and C. crustaceans. The figure introduces an enlargement of the Y axis of Figure 2 to better characterize the compound just below the chronic effect. The bar plots represent the sum of TU of the different

compounds at each site. Compounds with the largest contribution to the TU and compounds with higher contributions are named in this figure, all the other compounds' TU with minor contributions are summarised in the further compounds.

Table S4: Toxic Units of individual compounds for algae

Table S5: Toxic Units of individual compounds for crustacean

Table S6: Toxic Units of individual compounds for fish

Table S7: EC 50 of compounds

Section S5: Additional information on impacts on WWTP influent and effluent

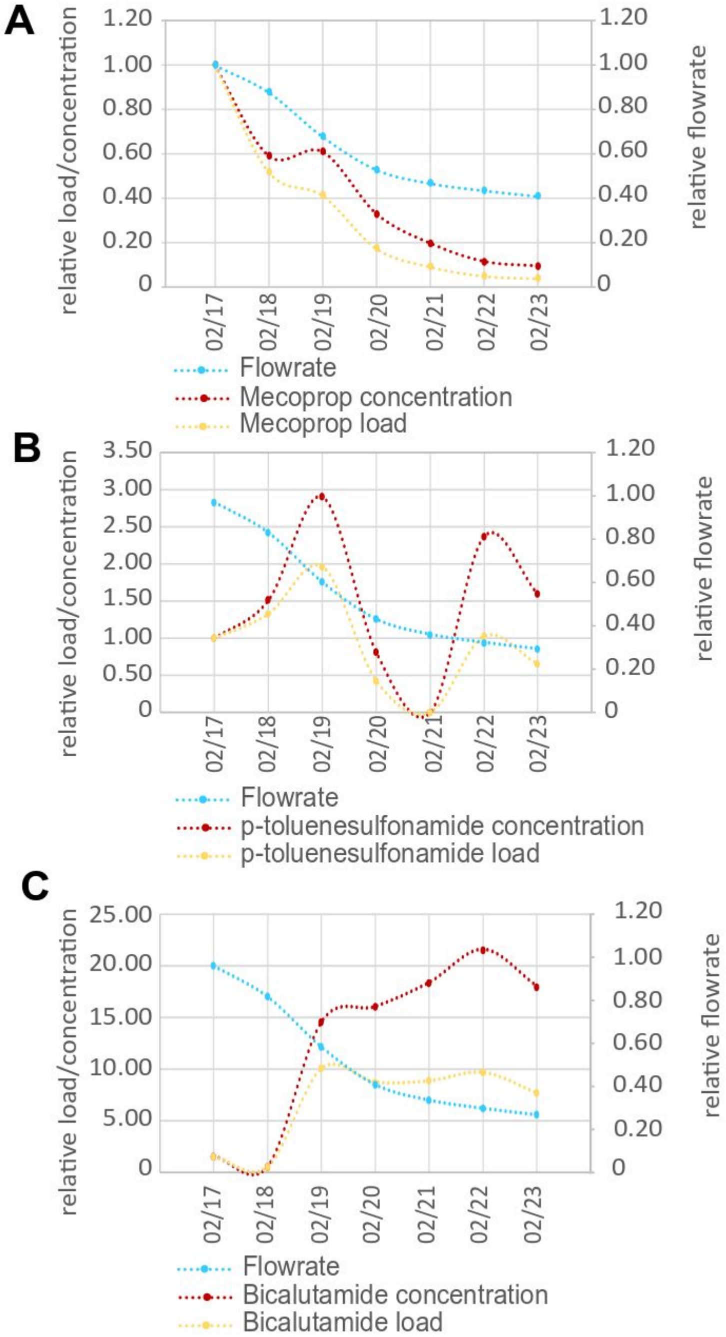

Figure S3 A Spearman correlation coefficient between compound concentrations and WWTP influent flow rate. The bar plots in red indicate a positive correlation and the ones in blue a negative correlation and (A;B;C) three examples of compound with temporal trends showing a strong positive, negative and no correlation with flow rate. The first date (February 17th) has been used as reference.

Table S8: Concentration in WWTP influent of chemical compounds found in the snow melt samples and the WWTP influent

Table S9: Concentration in WWTP effluent of chemical compounds found in the snow melt samples and the WWTP influent and effluent

Table S10: Discharges WWTP

Table S11: Compounds removal rate in WWTP

Supporting references

(1) Schulze, T.; Ahel, M.; Ahlheim, J.; Aït-Aïssa, S.; Brion, F.; Di Paolo, C.; Froment, J.; Hidasi, A. O.; Hollender, J.; Hollert, H.; Hu, M.; Kloß, A.; Koprivica, S.; Krauss, M.; Muz, M.; Oswald, P.; Petre, M.; Schollée, J. E.; Seiler, T.-B.; Shao, Y.; Slobodnik, J.; Sonavane, M.; Suter, M. J.-F.; Tollefsen, K. E.; Tousova, Z.; Walz, K.-H.; Brack, W. Assessment of a Novel Device for Onsite Integrative Large-Volume Solid Phase Extraction of Water Samples to Enable a Comprehensive Chemical and Effect-Based Analysis. *Science of The Total Environment* **2017**, *581–582*, 350–358. https://doi.org/10.1016/j.scitotenv.2016.12.140.

(2) Välitalo, P.; Massei, R.; Heiskanen, I.; Behnisch, P.; Brack, W.; Tindall, A. J.; Du Pasquier, D.; Küster, E.; Mikola, A.; Schulze, T.; Sillanpää, M. Effect-Based Assessment of Toxicity Removal during Wastewater Treatment. *Water Research* **2017**, *126*, 153–163. https://doi.org/10.1016/j.watres.2017.09.014.

(3) Kessner, D.; Chambers, M.; Burke, R.; Agus, D.; Mallick, P. ProteoWizard: Open Source Software for Rapid Proteomics Tools Development. *Bioinformatics* **2008**, *24* (21), 2534–2536. https://doi.org/10.1093/bioinformatics/btn323.

(4) Pluskal, T.; Castillo, S.; Villar-Briones, A.; Orešič, M. MZmine 2: Modular Framework for Processing, Visualizing, and Analyzing Mass Spectrometry-Based Molecular Profile Data. *BMC Bioinformatics* **2010**, *11* (1), 395. https://doi.org/10.1186/1471-2105-11-395.

(5) Beckers, L.-M.; Brack, W.; Dann, J. P.; Krauss, M.; Müller, E.; Schulze, T. Unraveling Longitudinal Pollution Patterns of Organic Micropollutants in a River by Non-Target Screening and Cluster Analysis. *Science of The Total Environment* **2020**, *727*, 138388. https://doi.org/10.1016/j.scitotenv.2020.138388.
